# The PCSK9 revolution and the potential of PCSK9-based therapies to reduce LDL-cholesterol

**DOI:** 10.21542/gcsp.2017.2

**Published:** 2017-03-31

**Authors:** Nabil G. Seidah

**Affiliations:** Laboratory of Biochemical Neuroendocrinology, IRCM; Affiliated to the University of Montreal, 110 Pine Avenue West, Montreal, QC, H2W 1R7 Canada

## Introduction

A large number of clinical trials over the last 30 years have firmly consolidated the importance of lowering low density lipoprotein cholesterol (LDLc) in the prevention of cardiovascular diseases (CVD) and its associated devastating sequelae.^1^ While healthy diets and exercise are highly recommended to lower LDLc levels, in many individuals with high baseline levels of LDLc, this is not sufficient to bring levels down to recommended target values in order to prevent recurrent coronary heart disease and cardiovascular complications. This is especially true for patients at high risk of premature cardiovascular death and disability, including those with familial hypercholesterolaemia (FH). FH is a very common inherited disease – affecting at least 30 million people worldwide, with an overall incidence of 1:200 globally^2^ – of whom ≤1% have been diagnosed. The advent of HMG-CoA reductase inhibitors, also known as “statins”, and their first application to hypercholesterolemic patients over 30 years ago, has revolutionized the treatment of FH patients and resulted in substantial lowering of LDLc. In addition, cholesterol–lowering drugs, such as “ezetimibe” that blocks cholesterol absorption from the gut by inhibiting the Niemann-Pick C1-like 1 (NPC1L1) transporter, have also been successful and a 7-year IMPROVE-IT trial revealed that a “simvastatin-ezetimibe” combination resulted in an incremental lowering of LDLc levels and a modest 2% improved cardiovascular outcomes.^3^ Therefore, it became clear that additional treatments are needed to substantially decrease LDLc and efficiently protect against CVD.

In 2003, the identification of the proprotein convertase subtilisin-kexin # 9, and the genetic evidence of its up-regulation of the levels of circulating LDLc^4,5^ *via* the enhanced degradation of the LDL receptor (LDLR)^6^, was an unexpected and welcome addition to the armamentarium of drug targets aimed to safely lower LDLc to levels never achieved before.^7,8^ Indeed, the discovery of PCSK9 and its induced-degradation of the LDLR revolutionized the field of LDLc-regulation. Amazingly, knowledge went from bench-to-bedside in less than nine years. A new PCSK9-targeted class of medicine is emerging, representing the biggest weapon against heart disease since the development of “statins”. The current crop of PCSK9 inhibitors are injectable monoclonal antibodies (mAb) to treat patients who cannot tolerate statins, or whose LDLc is not controlled by drugs. Food and Drug Administration approval of the first of a new class of therapeutics (PCSK9 mAb) was achieved in 2015. The present review will briefly describe the properties of PCSK9, our current understanding of its biology and intracellular trafficking, and then discuss the status of the various approaches that have been proposed to lower the levels of PCSK9.

## The *PCSK*-family of proprotein convertases subtilisin-kexin types

**Figure 1.**
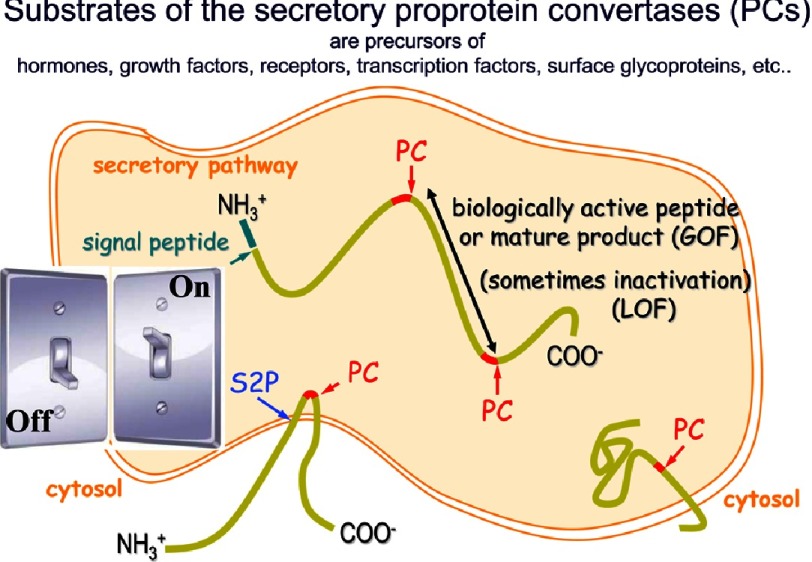
Schematic representation of the limited proteolysis of secretory precursor proteins. Notice that such PCSK-generated cleavages can either activate the cognate precursor by releasing bioactive products or inactivate it by removing bioactive moieties.

**Figure 2.**
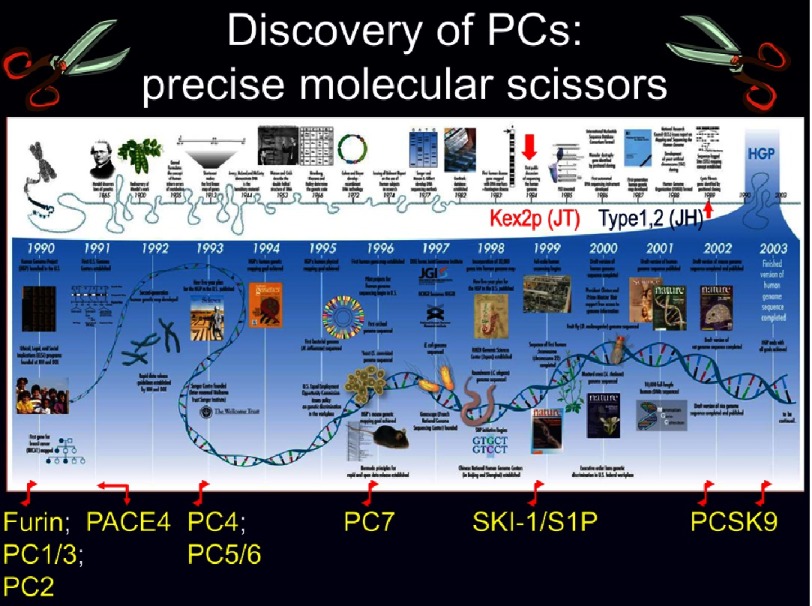
History of the discovery of the proprotein convertases. The first discovery of Kexin in 1984, led the way to the identification of its 9 mammalian homologues from 1990–2003.

**Figure 3.**
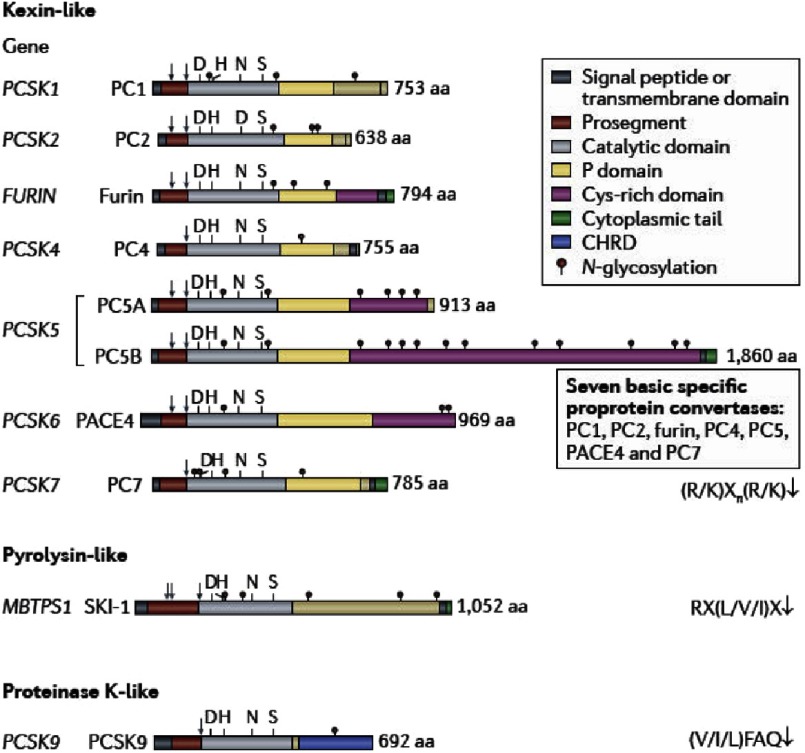
Schematic representation of the primary structures of the human proprotein convertases. The kexin-like basic amino acid (aa)-specific proprotein convertases, pyrolysin-like subtilisin kexin isozyme 1 (SKI-1; encoded by the MBTPS1 gene) and proteinase K-like proprotein convertase subtilisin kexin 9 (PCSK9) are individually grouped to emphasize their distinct subclasses. The various domains and N-glycosylation positions are emphasized, along with the primary (depicted using light grey arrows, and a light grey double arrow for SKI-1) as well as the secondary autocatalytic processing sites (depicted using dark grey arrows). The presence of a signal peptide, a prosegment and catalytic domain is common to all convertases that exhibit the typical catalytic triad residues Asp, His and Ser, as well as the Asn residue comprising the oxyanion hole (Asp for PC2). The carboxy-terminal domain of each convertase contains unique sequences regulating their cellular localization and trafficking. Thus, PCSK9 exhibits a Cys-His-rich domain (CHRD) that is required for the trafficking of the PCSK9–LDLR (low-density lipoprotein receptor) complex to endosomes and lysosomes. (modified from^11^).

It became clear from the mid-1960s that many eukaryotic secretory proteins were cleaved by proprotein convertases (PCs) to generate active peptide/protein products from their original inactive precursors.^9,10^ Cleavage occurred at specific exposed single or paired basic amino acid sites within the consensus motif (**K/R**)-X_0-6_-(**K/R**)↓, where Arg is the preferred residue at the P1 cleavage site over Lys (Figure 1).^11^ This involved a variety of precursors of polypeptide hormones, growth factors, receptors, enzymes, adhesion molecules, and even cell surface proteins from infectious viruses, parasites and bacteria. Such widespread precursor activation was found to occur in most species in both eukaryotes and even in some prokaryotes. However, it subsequently became apparent that such limited cellular proteolysis can also inactivate specific bioactive proteins (Figure 1).^12^ It took more than 15 years of intensive research by a number of teams in both North America and Europe to hunt for the elusive PCs, which were estimated to be present at <100-fold lower concentration than their substrates. Using powerful yeast genetics, the first successful identification of a PC was reported in 1984 for the processing of the precursor of *α*-mating factor (Figure 2).^13^ The enzyme, named “Kexin or Kex2p”, turned be an ancient serine protease related to the bacterial family of subtilisin-like proteases.^14,15^ The ability of Kexin to precisely process mammalian precursors at the expected physiological sites supported the hypothesis that Kexin is a prototype of the as yet unidentified mammalian proprotein convertases.^16^ The first glimpse of the properties of such mammalian proteinases was obtained in 1988 upon analysis of a human insulinoma tumor highly enriched in both hormone and its processing proteinases.^17^ It turned out that two such convertases (type 1 and 2) were needed for the generation of active human Insulin from its inactive precursor proInsulin. However, it was not until the advent of gene and cDNA cloning and expression that in 1990 the first 3 members, PC1,^18^ PC2,^18,19^ and Furin^20^ of the PC-family were finally identified, cloned and their activity validated in cells (Figure 2). The genes coding for these enzymes were named *PCSK1*, *PCSK2* and *Furin* (Figure 3). From 1990–1997 four more convertases were consecutively identified and cloned, giving a total of seven basic-residue-specific PCs (Figure 3).

The above enzymes differ in their tissue expression and subcellular localization.^21^ Briefly, the soluble PC1 and PC2 are found exclusively in dense-core secretory granules in endocrine and neural tissues, and are responsible for the activation of most polypeptide hormones.^12^ The type I-membrane-bound Furin and PC7 are ubiquitously expressed,^11^ and sometimes share similar precursor substrates such as those of Sortilin and Brain Derived Neurotropic Factor (proBDNF).^22^ The soluble PC5A and PACE4 are widely expressed and often activate cell surface precursors, such as cell surface receptors and growth factors.^12,23^ Animals completely lacking the convertases mouse Furin, and human and mouse PC5 have severe developmental defects, and they die before birth.^11,24,25^ In contrast, mice lacking PC7 are quite healthy, and are anxiolytic and novelty seekers.^22^ The various physiological and pathological functions of these 7 basic-residue-specific PCs have been extensively reviewed elsewhere^11,12,23,26^ and will not be examined any further in this paper.

In our search for other members of the *PCSK*-family, in 1999 we identified an eighth member that we called subtilisin-kexin isozyme 1 (SKI-1), because it was able to cleave proBDNF at a non-basic site within the recognition motif **R**-X-**L**/**V**/**I**-X↓ (Figure 3).^27,28^ This is also a type-I membrane bound protease best related to the pyrolysin family of subtilases^29^. Independently, it was also discovered that SKI-1 (also called site 1 protease) was responsible for the processing of various membrane-bound transcription factors, such as sterol regulatory element binding proteins (SREBPs)^30^ and the ER stress sensor ATF6.^31^ Therefore, SKI-1/S1P plays a major role in the regulation of lipogenic genes, including those of LDLR and PCSK9, as we shall see later. Because of these activities, the gene for SKI-1/S1P is now called Membrane-Bound Transcription Peptidase Site 1 (*MBTPS1*). Our extensive studies of the properties of this enzyme revealed that it undergoes a very unique autocatalytic activation, which is quite different from those of the other seven basic-residue specific PCs.^28,32,33^

In addition, this unique enzyme was also reported to activate the phosphorylation of mannose residues in proteins destined to lysosomes, since it is required to activate the α∕β-subunit precursor protein of the GlcNAc-1-phosphotransferase forming mannose 6-phosphate (M6P) targeting markers on lysosomal enzymes.^34^ Interestingly, this activity is independent of the lipogenic transcription control by cholesterol and fatty acids.^35^ SKI-1/S1P was also shown to be critical for neuronal axonal growth, ^36^ and for bone osteoblast mineralization.^37^ Thus, SKI-1/S1P may have other unsuspected functions in specific tissues that are independent of SREBPs or ATF6. Caudal regression syndrome (sacral agenesis), which impairs development of the caudal region of the body, occurs with a frequency of about 1 live birth per 50,000 newborns, although this incidence rises to 1 in 350 infants born to mothers with gestational diabetes.

The complete knockout of the mouse *Mbtps1* is embryonically lethal at very early developmental stages. Thus, to better understand the role of SKI-1/S1P in osteogenic differentiation and skeletal development, we used a tissue-specific approach to delete the expression of SKI-1/S1P in chondrocytes. This conditional *Mbtps1* loss-of-function mouse model exhibits phenotypic changes localized to the lumbar/sacral vertebral region (decreased vertebral number, vertebral fusion, and kinky tail) that mimic those in caudal regression syndrome, suggesting that loss-of-function mutations in *Mbtps1* may cause the etiology of this disease.^38^

## The discovery of PCSK9 and its genetic relationship to LDL

**Figure 4.**
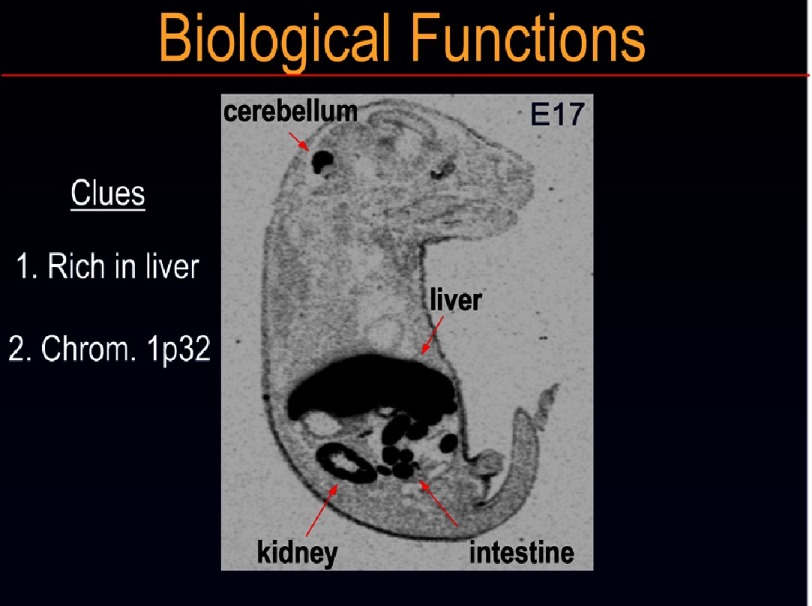
In situ hybridization histochemistry of the expression of PCSK9 mRNA in an embryonic day 17 (E17) mouse. Notice the high expression of PCSK9 in liver, small intestine, kidney and cerebellum. The locus of the human *PCSK9* gene on the small arm of chromosome 1p32 is emphasized. This pattern of tissue expression of PCSK9 was the basis that led us to define its role in liver in cholesterol regulation.

**Figure 5.**
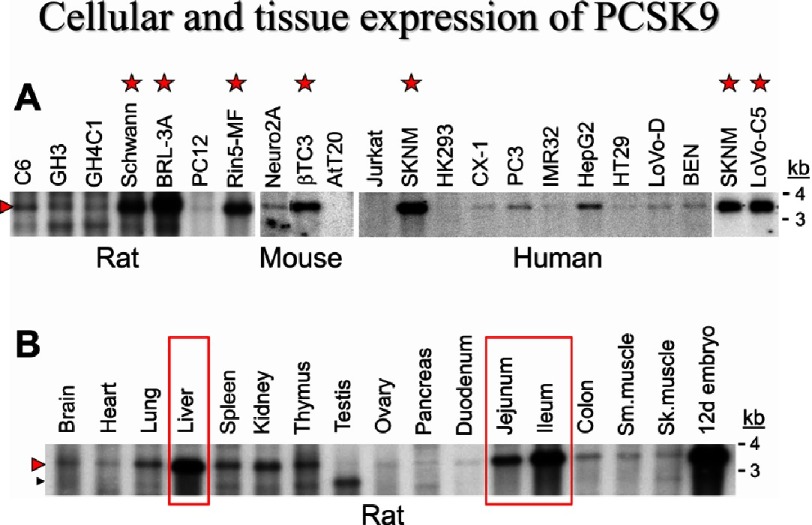
Cellular and tissue expression of PCSK9. Northern blot analysis of PCSK9 mRNA in 21 rat, mouse, and human cell lines (A) and 17 rat tissues (B) (Sm, smooth; Sk, skeletal; d, day). The open arrow points to the smaller testicular mRNA. The red stars denote high expression of PCSK9 in specific tumor derived cell lines. The tissues expressing high levels of PCSK9 are boxed in. Modified from.^5^.

During an exhaustive PCR-based homology search to the *PCSKs*, in mouse, rat and human cell lines in 2002 (reported in early 2003), ^5^ we cloned a novel cDNA sequence encoding a 24–25% identical catalytic subunit (260 aa) to the subtilases SKI-1/S1P, PC7, and tripeptidyl peptidase II. Using a protein BLAST program (www.ncbi.nlm.nih.gov/BLAST), similar sequences were identified in patented databases (Millennium Pharmaceuticals, Cambridge, MA, patent no. WO 01/57081 A2; and Eli Lilly, LP251 patent no. WO 02/14358 A2). The sequence identified by Millennium was obtained following serum deprivation in primary cerebellar neurons leading to the development of apoptosis. Thus, the gene product was originally called Neural Apoptosis Regulated Convertase 1 (NARC-1).^5^ Upon inspection of the putative protein sequence we immediately realized that it encoded a new ninth member of the *PCSK*-family of proprotein convertases (now called PCSK9) and showed that it localized to human chromosome 1p32. Not knowing what the function of the enzyme was, we first defined its tissue and cellular distribution and showed that it was highly enriched in the adult liver, small intestine, kidney cortex and cerebellum (Figure 4).^5^ *In situ* hybridization and Northern blot analyses of PCSK9 expression during development, and in the adult, and after partial hepatectomy, revealed that it is expressed in cells that have the capacity to proliferate and differentiate. These include hepatocytes, kidney mesenchymal cells, intestinal ileum, and colon epithelia as well as embryonic brain telencephalon neurons.^5^ It was also highly expressed in various tumor-derived cell lines (Figure 5).

The localization of the *PCSK9* gene on the short arm of chromosome 1p32, led us to initiate a very fruitful collaboration with Catherine Boileau and Marianne Abifadel in Paris, who were on the lookout for a gene located on chromosome 1p32-p34.1, which by linkage analysis was thought to represent the third FH locus (FH-3) different from the LDLR (FH-1) and its ligand apoB (FH-2). It turned out that indeed the gene coding for PCSK9 is the one responsible for observed hypercholesterolemia phenotype observed in two French families, with the rare gain-of-function (GOF) mutations S127R and F216L of human PCSK9.^4^ For a more extensive review of the huge amount of detective work that led to this conclusion, the reader is referred to an excellent review of the history of the identification of PCSK9 as the third FH locus from their genetic perspective.^39^

**Figure 6.**
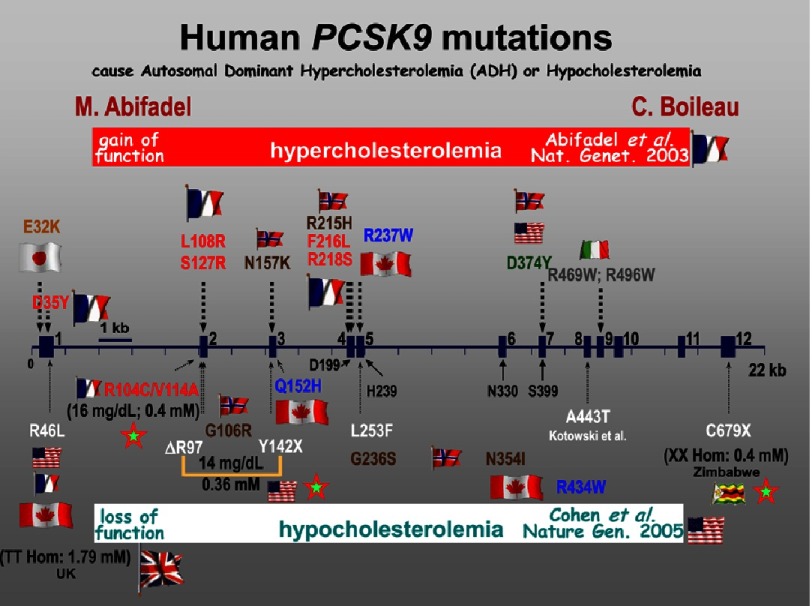
Schematic representation of the exon/intron structure of the PCSK9 gene and the identified GOF and LOF mutations. The first reports of GOF (above the gene) and LOF (below the gene) PCSK9 mutations are emphasized, as well as the countries of origin of each reported mutation. Notice that the 3 complete LOF mutations of PCSK9 are denoted with green stars with a red border, and the 0.4 mM levels of LDLc reached.

In essence, the two GOF point mutations in PCSK9 that were identified in the two French families (from Nantes and Bordeaux) were responsible for the 2-fold (F216L) and 4-fold (S127R) increase of circulating LDLc in these FH-3 patients (Figure 6). Since this seminal discovery, a number of rare missense GOF mutations in each of the 12 PCSK9 exons were identified, always leading to higher levels of LDLc (Figure 6). The most damaging one is the Anglo-Saxon mutation D374Y, occurring in exon 7, first identified in the Mormon Population in Utah,^40^ and later on found in England and other countries.^7^ The LDLc in these heterozygous patients is at least 5-fold higher than normal. The GOF D374Y-PCSK9 causes a severe FH phenotype that is not readily reduced by statins.^41^ Carriers of this mutation are typically affected by CVD 10 years earlier than other FH patients.

On the other hand, the more common loss-of-function (LOF) mutations in PCSK9 result in low levels of circulating LDLc (Figure 6).^42^ In fact, complete heterozygote LOF of PCSK9 was estimated to result in an 88% reduced risk of developing cardiovascular complications over a 15-year follow-up period.^43^ Another example is the R46L mutation, which results in a partial LOF, and is associated with a 15% reduction in LDLc, and a 47% reduction in the risk of coronary heart disease.^43^ Amazingly, two women and one man were identified who completely lacked PCSK9 expression, and all have similarly low levels of LDLc of 0.4 mM, which is about 7–8-fold lower than normal (Figure 6). These included: (**1**) an African American woman presenting compound heterozygote PCSK9 LOF deletion/truncation mutations (ΔR97 + Y142X), apparently in good health;^44^ (**2**) An African woman from Zimbabwe that presented a homozygote LOF truncation C679X;^45^ and (**3**) A French diabetic Caucasian that presents compound LOF mutations (R104C +V114A).^46^ These results, and the observation that patients exhibiting LOF PCSK9 mutations have an increased LDLc catabolic rate, ^46^ with apparently no obvious deleterious effects associated with very low LDLc levels, provided compelling arguments for developing inhibitors of PCSK9 to treat hypercholesterolemia.

## The cellular biology of PCSK9 and its trafficking

However, before doing so, it was necessary to understand the mechanism by which high concentrations of PCSK9 or GOF mutations are associated with high LDLc levels, with the reverse being true for LOF mutations. Indeed, epidemiological studies suggested that plasma PCSK9 levels correlate with high LDLc levels,^47–49^ suggesting a causal relationship.

**Figure 7.**
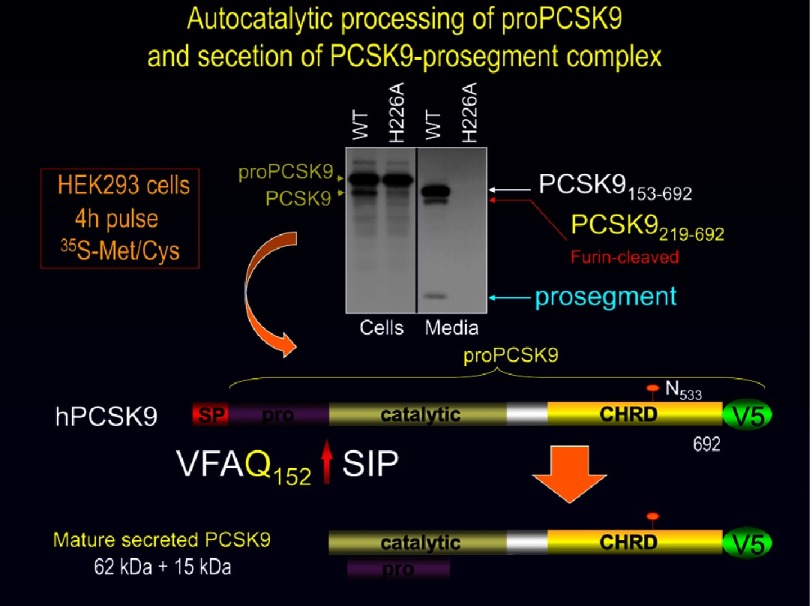
Biosynthesis of PCSK9 in HK293 cells. The V5-tagged PCSK9 or its active site mutant H226A were transiently expressed in HEK293 cells. The next day the cells were pulse-labeled with [^35^S]Met/Cys for 4 h. Cell extracts (C) and media (M) were immunoprecipitated with a V5 antibody and the precipitates were resolved by SDS/PAGE on an 8% tricine gel. The migration positions of molecular mass standards (kDa), proPCSK9, PCSK9 and the prosegment are emphasized, together with the Furin cleaved product observed. Note that the active site mutant is neither cleaved not secreted, emphasizing the necessity of prodomain autocatalytic cleavage for PCSK9 to exit the ER and be secreted.

**Figure 8.**
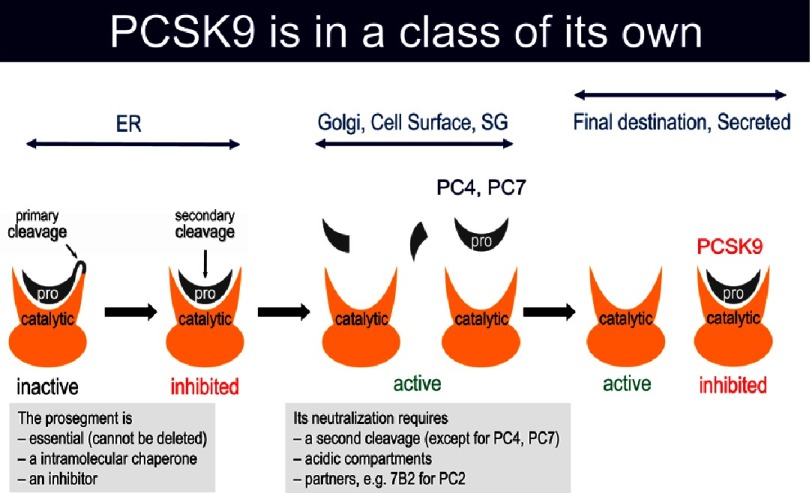
Zymogen activation of the proprotein convertases. The various strategies used by the convertases to get activated. It all starts in the ER where the first autocatalytic cleavage occurs. Except for PCSK9 all the other convertases get rid of their inhibitory prodomain to become enzymatically active.

Like all of the other seven proprotein convertases (with the exception of PC2 that has its own chaperone 7B2) PCSK9 undergoes an autocatalytic cleavage of its inhibitory prodomain at the VFAQ_152_↓ site in the endoplasmic reticulum (ER), resulting in tightly-bound heterodimer of the prosegment and the rest of the mature protein, ^5,50^ that in this state is proteolytically inactive.^23^ Such zymogen cleavage allows the protein to exit the ER and traffic though the Golgi apparatus and is secreted within minutes (Figure 7).^50^ However, PCSK9 is in a class of its own, since it always remains in a tight enzymatically inactive complex with its prodomain (Figure 8) unlike other PCSKs that lose their prodomain following a second cleavage or dissociation of the prodomain along their intracellular route before reaching their final destinations. Indeed, the crystal structure of secreted mature PCSK9 confirmed these biochemical observations and revealed the very tight association of the prodomain with the catalytic subunit of PCSK9.^51^ These data indicated that the circulating PCSK9 is enzymatically inactive due to its association with the inhibitory prodomain.

However, the identification of GOF mutations F216L and R218S led to the demonstration that these PCSK9 mutations are associated with the loss of the ability of another proprotein convertase – Furin – to cleave and inactivate PCSK9 at the sequence **R**FH**R**_218_↓.^52,53^ This results in the dissociation of the prodomain and segment 153–218, resulting in a truncated inactive form of PCSK9.^52^ This new concept suggested that PCSK9 is subject to inactivation prior to its secretion from the liver (its main source in the plasma). Indeed, recent mass spectrometry evidence revealed that up to 40% of circulating PCSK9 is Furin-inactivated.^54^ This raises the question of the pertinence of measuring the circulating levels of PCSK9 using a simple ELISA that recognizes both active and inactive forms, and may explain the seemingly low level of correlation between circulating total concentrations of PCSK9 and LDLc.^48^ It is expected that the concentrations of the active form of PCSK9 would better correlate with those of circulating LDLc.

**Figure 9.**
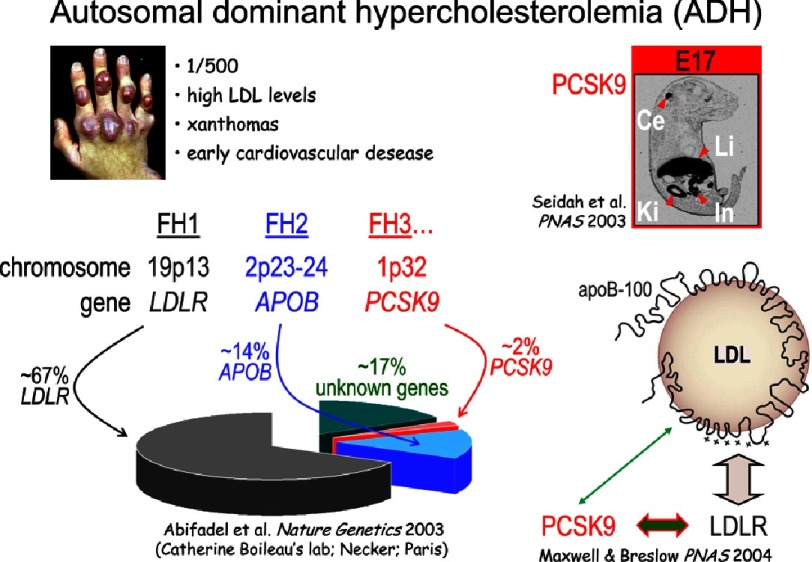
Schematic representation of the incidence of FH-1, 2 and 3 mutations and a model emphasizing the mutual binding of the LDLR, apoB and PCSK9. The tendon xanthomas seen in FH patients are shown on the top left corner.

The first indication of the mechanism underlying the observed PCSK9 regulation of LDLc was reported by Maxwell and Breslow, when they demonstrated that PCSK9 targets the LDLR towards an intracellular degradation compartment^6^ We showed that this degradation occurs in an acidic compartment, and that it involves endocytosis of the cell surface PCSK9 ≡ LDLR complex into clathrin heavy chain coated early endosomes.^55^ It was later shown by the group of Horton and Hobbs that the catalytic domain of PCSK9 binds the EGF-A domain of the LDLR, ^56^ and that the enzymatic activity of PCSK9 is not necessary for its induced degradation of the LDLR^57^ Interestingly, the GOF PCSK9-D374Y mutant was found to bind to the LDLR with a 6- to 30-fold higher affinity compared with wild-type PCSK9, by reinforcing a hydrogen bond between PCSK9 and the EGFA domain of the LDLR.^51^ In fact, it seems that the negative charge of Asp_374_ is the critical negative factor, as it can be replaced by Glu_374_, but its replacement by uncharged or hydrophobic residues results in similar GOF as Tyr_374_.^58^ It is now well accepted that the bioactive heterodimeric prodomain ≡ PCSK9 binds the EGF-A domain of the LDLR,^59^ and the trimeric prodomain ≡ PCSK9 ≡ LDLR complex is escorted to endosomes/lysosomes for degradation, but that the underlying details of the trafficking mechanism remain obscure.^7^ Therefore, the 3 FH genes interact with each other as PCSK9 binds both the LDLR^6^ and apparently apoB^60^ (Figure 9).

However, we consistently observed that mutants of LDLR (e.g., LDLR-L339D) that cannot be targeted to endosomes/lysosomes by extracellular PCSK9, are still degraded intracellularly in various cell lines by co-expressed PCSK9. This led to the discovery of an intracellular pathway of PCSK9-induced LDLR degradation that does not require PCSK9 secretion and that drags the LDLR to lysosomes directly after exit of the trimeric prodomain ≡ PCSK9 ≡ LDLR complex from the trans-Golgi network into clathrin light chain coated endosomes.^61^ Such a distinct intracellular pathway, while prevalent in most cell lines transfected with wild type PCSK9 (but not its D374Y mutant), does not seem to play a major role in the PCSK9-enhanced degradation of the LDLR in liver hepatocytes *in vivo*. The reason for this discrepancy between liver and hepatocyte cell lines is still not clear.^7^

Genetic and cellular evidence revealed that the phospho-tyrosine binding protein ARH, which recognizes **NP**X**Y** motifs in cytosolic tails (CT) of membrane-bound proteins, is required for the internalization of the LDLR-PCSK9 complex into clathrin-coated vesicles, leading to the enhanced degradation of the complex by the extracellular pathway^62^ However, the CT of the LDLR containing an **NP**X**Y** motif is not needed for the internalization of the PCSK9 ≡ LDLR complex^63,64^, and we recently obtained evidence that even a soluble form of the LDLR lacking its transmembrane (TM) and CT domains (sLDLR) is still well targeted by PCSK9 for intracellular degradation. This suggests that one or more, as yet unidentified, protein(s) (X-protein; Xp), which contains a TM and one or more **NP**X**Y** motifs binds the cell surface PCSK9 ≡ LDLR complex and directs it to endosomes/lysosomes. In that context, we recently reported that the two proposed candidate Xps to chaperone the PCSK9 ≡ LDLR complex to degradation via the extracellular pathway, i.e., Sortilin (SORT1) and the amyloid β-precursor-like protein 2 (APLP2), do not physiologically regulate PCSK9 in cells and *in vivo*.^65^ Furthermore, our study eliminated them as candidate sec24a-binding proteins which were seemingly required for PCSK9 to exit from the ER into COP-II vesicles.^66^ Therefore, the mechanistic details and the trafficking components that regulate both degradation pathways of the PCSK9 ≡ LDLR complex are still unknown.

## Inhibition of PCSK9 reduces LDLc levels & incidence of CVD

Using animal models, it became clear that knockout of the *Pcsk9* gene in mice results in a hypocholesterolemia phenotype with an 80% reduction in LDLc,^67,68^ an enhanced response to statins^67^, and a significant decrease in the development of atherosclerosis^69^ The reverse is observed in transgenic mice overexpressing the wild type form of PCSK9,^69^ or its D374Y GOF mutant,^70^ and in transgenic pigs expressing the D374Y mutant.^71,72^

Some other benefits that result from the loss of PCSK9 expression are the reduced incidence of inflammation,^7^ sepsis,^73^ and tumor metastasis^74^ In addition, the observed aorta and vascular calcification in FH patients was reproduced in *Ldlr* KO mice and *Pcsk9* KO are protected, while PCSK9 overexpression exacerbates the phenotype.^75^ This is in part due to an inflammatory response to the formation of cholesterol crystals in hypercholesterolemic conditions that can be reversed by the administration of anti-inflammatory mAbs to Interleukin-1β.^76^

**Figure 10.**
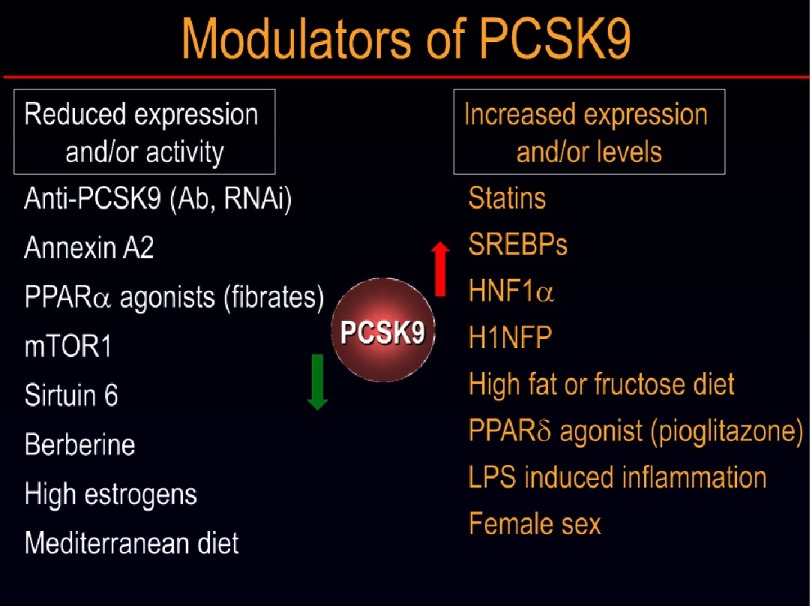
Modulators of PCSK9 function or expression. The activators are denoted with a red arrow, whereas inhibitors of PCSK9 are on the left side of the green arrow.

Analysis of various reagents that modulate PCSK9 levels (Figure 10), revealed that HNF1*α*^77^ is the strongest activator of PCSK9 transcription, while SREBP-2 upregulates the expression of both the LDLR and PCSK9.^47^ The latter regulation explains why statins, inhibitors of cholesterol synthesis, also activate the production of both PCSK9 and its target LDLR.^47^ In fact, it was recently reported that some cholesterol ester transfer protein (CETP) small molecule inhibitors that enhance the levels of HDL can also inhibit SREBP-2 and hence PCSK9 transcription, resulting in reduced LDLc.^78^ Mediterranean diet as well as estrogens and mTOR1 all reduce the levels of PCSK9, while inflammation and high fat or fructose rich diets^79^ increase the levels of PCSK9.^7^

**Figure 11.**
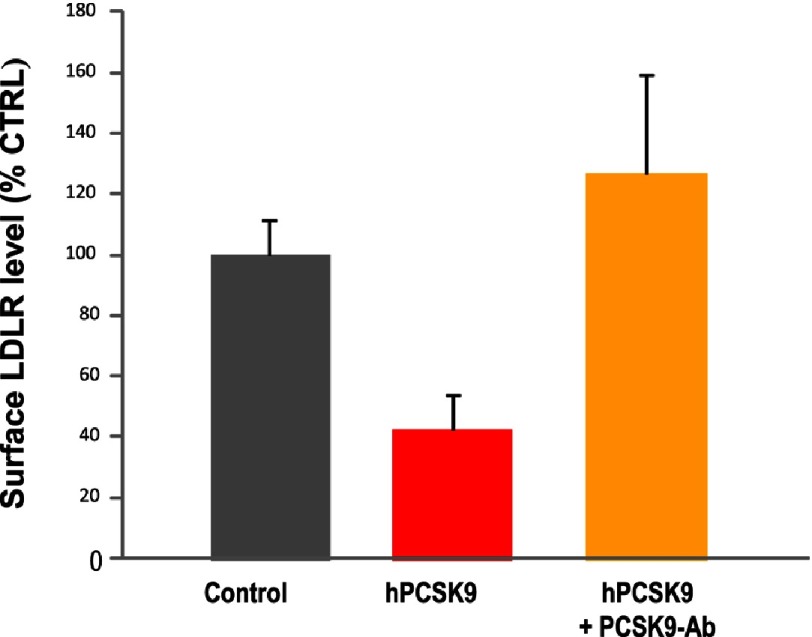
Effect of affinity purified PCSK9 polyclonal antibody on LDLR levels in HepG2 cells. Human PCSK9 (0.6 µg) was pre-incubated at neutral pH and 37°C for 1 h with saline control or 5 µg of a previously reported affinity-purified polyclonal antibody to PCSK9.^55^ These solutions were then incubated with HepG2 cells for 6 h, following which the cells were suspended and immediately analyzed by FACS for surface human LDLR levels.^58^ Notice that PCSK9 reduces the cell surface LDLR by >60%, and that the PCSK9 antibody completely reverses this activity on the LDLR.

However, the most powerful PCSK9 inhibitors are antibodies that prevent the binding of extracellular (circulating) PCSK9 with the LDLR. The polyclonal antibodies isolated in our group in 2007, ^55,80^ were found to be good inhibitors of the extracellular PCSK9 function on LDLR in HepG2 cells stably expressing PCSK9 (Figure 11). Other polyclonal antibodies that have a similar inhibitory function were also reported by N. Hooper’s group in 2009.^81^ The first proof of concept that a fully humanized PCSK9-mAb can effectively inhibit its function on LDLR *via* an allosteric mechanism and reduce LDLc levels in mice and monkeys was reported in 2009.^82^ Since then, at least three pharmaceutical companies have developed humanized mAbs against PCSK9, evolcumab, alirocumab and bococizumab, and all are in phase III clinical trials, of which outcomes are expected to become public by 2018.^7,83^

## Proposed strategies to inhibit PCSK9 function

Multiple studies in cells, animals and human LOF mutations revealed that PCSK9 inhibition would increase the levels of the LDLR in liver and hence reduce those of circulating LDLc. Such a mechanism of LDLc lowering is distinct and complementary to that of statins that inhibit cholesterol synthesis, resulting in the activation of SREBP-2 and consequently upregulating the mRNA levels of the LDLR, but also those of PCSK9 that reduces LDLR protein concentrations. Clearly, inhibition of PCSK9 would maximize the effect of statins and result in a drastic reduction of LDLc.^84^ This hypothesis was confirmed by the application of multiple inhibitory strategies discussed below. However, the question arose whether PCSK9 inhibition should be reversible in case of unexpected secondary effects, or should it be permanent , as two of the three persons lacking PCSK9 due to complete LOF mutations seem to be in relatively good health. However, the fact that only a very limited number of people exhibit complete loss of PCSK9, and yet the complete cardioprotective LOF C672X heterozygote mutation is found quite frequently in black Africans (3.3% overall), but varied significantly among ethnic groups, ranging from 0% to 6.9%,^85^ begs for caution in the use of a permanent inhibition strategy.

**Figure 12.**
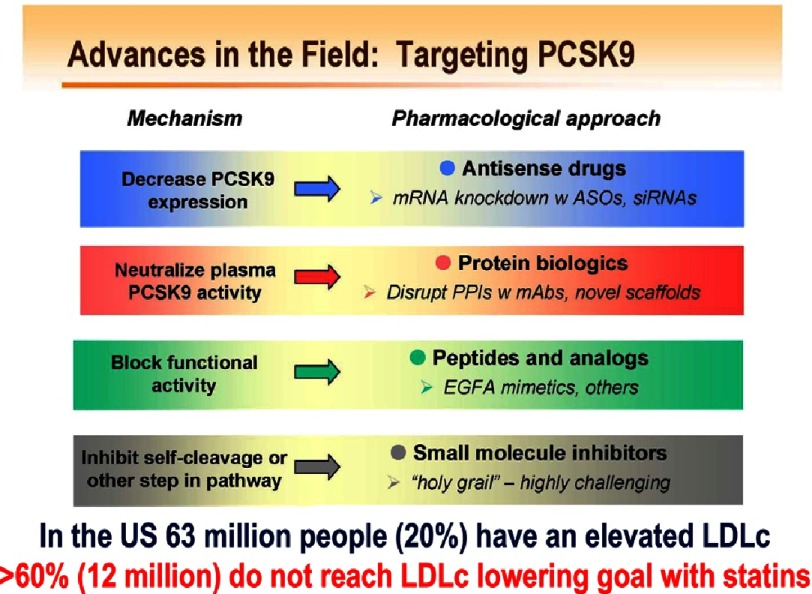
Various strategies to inhibit PCSK9 function or levels.

At least four general reversible strategies were proposed to target PCSK9 in order to reduce its circulating levels (Figure 12):

### 1. Neutralize plasma PCSK9 activity

#### mAb approach

**Figure 13.**
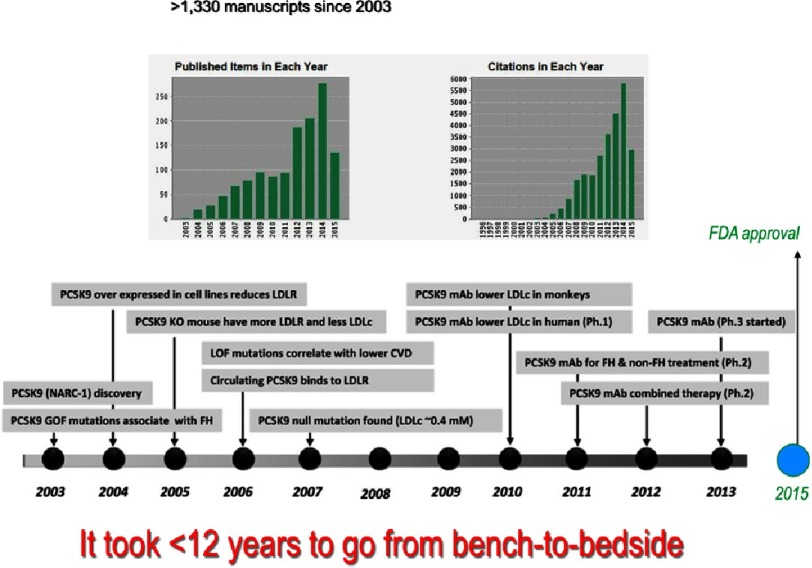
Proprotein convertase subtilisin kexin 9 (PCSK9): historical perspective from its discovery to clinical applications and the manuscripts associated with PCSK9 since 2003. The pace of research, from PCSK9 discovery through to clinical trials, has been rapid starting from its discovery in 2003 and the proof-of-principle that mAbs can inhibit its function in 2010, all the way to ongoing phase III clinical trials and the final approval by the FDA in 2015. CVD indicates cardiovascular disease; FH, familial hypercholesterolemia; GOF, gain-of-function; KO, knockout; LDL-C, low-density lipoprotein-cholesterol; LDLR, LDL receptor; LOF, loss-of-function. Notice that >1,330 manuscripts were published on the subject of PCSK9 since 2003, resulting in an H-index of 76.

The most advanced and proof-of-concept approach is definitively the use of inhibitory mAbs that allosterically deform the structure of the catalytic subunit of PCSK9, thereby preventing it from interacting with the EGF-A domain of the LDLR. The rapid progression of the knowledge and applications of PCSK9 inhibitors took <12 years and resulted in more than 1,300 publications (Figure 13). It is clear that the ‘injection every two-weeks’ or ‘once every month’ of an inhibitory mAb approach is the most advanced and privileged one today,^86^ as Evolocumab (Amgen) and Alirocumab (Sanofi/Regeneron) have been approved by the FDA and should be on the market by the end of 2015 or beginning of 2016. The use of the mAb Bococizumab (Pfizer)^87^ should follow shortly thereafter. The subcutaneous injection of these mAbs results in 50–60% reduction of LDLc and a 30–35% reduction in the highly atherogenic Lp(a) particles.^7,86,88^ The unexpected reduction in Lp(a) levels was recently rationalized by the fact that under supra-physiological levels of the LDLR, such as is the case with the use of inhibitory mAbs against PCSK9, the LDLR is the receptor of Lp(a).^89^

#### Adnectin approach

Adnectins are a family of binding proteins derived from the 10th type III domain of human fibronectin (10Fn3), which is part of the immunoglobulin superfamily and normally binds integrin. The 10Fn3 has the potential for broad therapeutic applications given its structural stability, ability to be manipulated, and its abundance in the human body and lack of immune response. Screening phage libraries for PCSK9 binders identified and led to the engineering of a high-affinity PCSK9 binder, called BMS-962476, as a potential alternative to mAbs. This is a ∼11-kDa polypeptide conjugated to polyethylene glycol to enhance pharmacokinetics, which binds with sub-nanomolar affinity to the catalytic subunit of human PCSK9, thereby inhibiting its interaction with the LDLR.^90^ In cynomolgus monkeys a 5 mg/kg single injection of BMS-962476, led to a ∼50% reduction of LDLc. This was accompanied by the reduction of circulating free PCSK9 levels by ∼6- to 7-fold over baseline, which then returned to control levels by 3 weeks, in parallel with return to baseline of LDLc levels, likely as a consequence of the Adnectin complex dissociating over time.^90^ We are awaiting the progress of this type of inhibitor in clinical trials.

### 2. Decrease PCSK9 expression

#### siRNA approach

One way to reversibly decrease PCSK9 expression would be to lower the levels of its mRNA. A small interfering RNA (siRNA; Alnylam Pharmaceuticals, Inc.) clinical trial involving siRNA-targeting PCSK9 has been evaluated in a randomized, single-blind, placebo-controlled, phase 1 dose-escalation study in healthy adult volunteers with serum LDLc of ≥3 mmol/L or higher.^91^ The data showed that at a dose of 0.4 mg/kg, this relatively safe treatment resulted in a mean 70% reduction in circulating PCSK9 plasma protein and a mean 40% reduction in LDLc from baseline relative to placebo. This siRNA approach was shown to be generally safe and well tolerated in this Phase I study and there were no serious adverse events related to study drug administration. Phase II clinical trials are underway. Although mAbs seem to block close to 100% of free circulating PCSK9, the siRNA approach left ≈30% PCSK9 in circulation, suggesting limited efficacy of the current siRNA method. Although a direct comparison of this approach with the mAb one is yet to be tested in humans, the efficacy of the reduction of LDLc observed with siRNA (40%)^91^ is still no better than that achieved with mAbs (50%–70%).^7,86^ This suggests that the intracellular pathway in liver may have a relatively minor contribution to the overall activity of PCSK9 on LDLR, which seems to mostly act by the extracellular pathway.

#### Transcriptional inhibition

*PCSK9* gene expression is regulated by SREBP-2, HNF1*α* and other factors (Figure 10).^7^ Thus, it is plausible to develop small molecules that would enter the nucleus in liver hepatocytes and decrease the transcription of the *PCSK9* gene. Interestingly, Kowa Pharmaceuticals reported that a CETP inhibitor (K-312) that raises HDL levels, and also inhibits PCSK9 transcription and lowers LDLc levels in rabbits. In the human hepatocyte-derived HepG2 cells, K-312 treatment decreased the active nuclear forms of SREBP-1 and SREBP-2 that regulate promoter activity of PCSK9. This suggests that K-312 may regulate the SKI-1/S1P or S2P cleavage and generation of the active nuclear forms of these SREBPs.^11^ Thus, K-312 decreases LDLc and PCSK9 levels, possibly serving as a new oral therapy for dyslipidemia and CVD. However, the SREBP target of this inhibitor makes it relatively non-specific for PCSK9, as the levels of other proteins regulated by SREBPs will also be affected.

#### Benzofurans as modulators of CVD

Tribbles pseudokinase 1 (TRIB1) is implicated in modulating the risk of CVD.^92^ Active benzofurans, as well as natural products capable of TRIB1 upregulation, also modulate hepatic cell cholesterol metabolism by elevating the expression of LDLR mRNA and LDLR protein, while reducing the levels of PCSK9 mRNA and secreted PCSK9 protein and stimulating LDLc uptake.^92^ The effects of benzofurans are not masked by cholesterol depletion and are independent of the SREBP-2 regulatory circuit, indicating that these compounds represent a novel class of chemically tractable small-molecule modulators that shift cellular lipoprotein metabolism in HepG2 cells from lipogenesis to scavenging. Time will tell if such an approach, that is not specific for PCSK9, is nevertheless safe and feasible clinically.

### 3. Block PCSK9 functional activity on the LDLR

#### EGF-A mimetics

Since PCSK9 interacts with the EGF-A domain of the LDLR, it is plausible that a competitive EGF-A mimetic can act as a decoy to block the activity of extracellular PCSK9 on the LDLR. The interaction surface of EGF-A and PCSK9 is large and flat, with the two proteins interacting *via* a 530 Å^2^ flat contact patch between the catalytic domain of PCSK9 and the EGF-A domain in the LDL-R.^59,93^ it is thus a real challenge to find a small molecule that would inhibit LDLR binding. Nevertheless, a number of approaches using peptidomimetics of EGF-A have been reported. Two truncated and modified versions of the EGF-A peptide were designed and found to be active in the low µM range to inhibit the activity of extracellular PCSK9 on the LDLR.^94,95^ Screens of phage-displayed peptide libraries led to the identification and synthesis of a 13-amino acid linear peptide (Pep2-8) as the smallest PCSK9 inhibitor known.^95^ However, much work is still necessary to stabilize such structures for *in vivo* applications, and possibly their transformation into orally active compounds.

#### Peptide mimetics of natural inhibitors of PCSK9

The search for natural inhibitors of PCSK9 led to the identification of Annexin A2 as a candidate inhibitor of the extracellular activity of PCSK9.^96,97^ The inhibitory domain was localized to be in the 70 aa N-terminal R1-repeat domain of Annexin A2, and this was used to develop a high nM PCSK9-inhibitor that is active in cell lines.^97^ Interestingly, Annexin A2 is a cytosolic protein that is secreted and meets PCSK9 in the extracellular milieu. Recent data also revealed that the cytosolic form of Annexin A2 also reduces PCSK9 protein levels *via* inhibition of its translation, likely upon binding inhibitory motifs in the 3′ untranslated region of the PCSK9 mRNA.^98^ The identification of the binding domain of cytosolic Annexin A2 to the PCSK9 mRNA may lead to the synthesis of intracellular inhibitors of PCSK9 translation.

### 4. Inhibit proPCSK9 zymogen autocatalytic cleavage or secretion from cells

The precursor proPCSK9 oligomerizes in the ER in a disulfide dependent fashion,^5^ and the exit of the monomeric prodomain ≡ PCSK9 from the ER (ultimately leading to secretion) is dependent on the zymogen autocatalytic processing of proPCSK9 into PCSK9 to excise the prodomain (Figure 1).^5,50^ It is thus plausible to identify an inhibitor, possibly a small molecule, that would prevent PCSK9 from exiting the ER, either by inhibiting its autocatalytic processing, or by enhancing its oligomerization. A number of screens have been initiated to inhibit autocatalytic processing of PCSK9. Since this is an *in cis* zero kinetics reaction it would be rather difficult, but not impossible, to block it. Indeed, novel assays have been proposed to test potential inhibitors of proPCSK9 processing, and suggested that the PCSK9 active site and its adjacent residues serve as an allosteric modulator of protein secretion, independent of its role in proteolysis, revealing a new strategy for intracellular PCSK9 inhibition.^99^

At least two permanent PCSK9 inhibition strategies have been proposed:

#### PCSK9 vaccination

A recent report suggested the use of peptide-based anti-PCSK9 vaccines, isolated *via* the generation of polyclonal high affinity and persistent antibodies that are functional for up to one year (AFFiRiS AG, Austria).^100^ In mice, they are reported to be powerful with an up to ∼50% reduction of LDLc and ∼30% decrease in total cholesterol. It was suggested that this type of vaccine is a safe tool for long-term LDLc management, and thus may represent a novel therapeutic approach for the prevention and/or treatment of hypercholesterolemia-related CVD in humans. However, the potentially negative long term consequences associated with this approach should not be underestimated, as it is also apparent that the permanent lack of PCSK9 may reduce the ability of the liver to regenerate^68^ and may enhance viral infections.^101,102^ Furthermore, the extreme rarity of individuals that completely lack PCSK9 is if anything, an indication of a potential counter-selection against this event during evolution. Notwithstanding these caveats, Pfizer is testing an experimental PCSK9 vaccine, designed to induce the body to produce its own PCSK9 antibodies, which should enter human testing in 2016. It is reported that that, if successful, the vaccine might eventually be an annual injection.

#### CRISPR-Cas9 gene silencing of PCSK9

Scientists hoping to alter the genome of their favorite organisms faced an arduous task, which has been vastly improved by the ability to quickly destroy or edit a gene with a new technology called CRISPR (clustered regularly interspaced short palindromic repeat)/Cas9. Such RNA-guided endonuclease Cas9 has emerged as a versatile genome-editing platform.^103–105^ In this method the Cas9 enzyme cuts DNA at a specific sequence, determined by an accompanying bit of RNA called a guide RNA. Then, the cell’s own DNA repair machinery typically takes over in one of two different repair modes: (**1**) it simply glues the two pieces back together, but imperfectly, so the leftover scar interrupts and disables the targeted gene; or (**2**) the cell can copy a nearby piece of DNA to fill in the missing sequence. By providing their own DNA template, scientists can now induce the cell to fill in any desired sequence, from a small mutation to a whole new gene.

A recent proof-of-principle study suggested the possibility of permanent alteration of PCSK9 with *in vivo* use of CRISPR-Cas9 genome editing.^106^ Here the authors injected mice with an adenovirus expressing CRISPR-Cas9 and a CRISPR guide RNA targeting Pcsk9 in mouse liver. The data showed that in 3–4 days from the administration of the virus, the mutagenesis rate of *Pcsk9* in the liver was >50%. This resulted in decreased plasma PCSK9 levels, increased hepatic low-density lipoprotein receptor levels, and decreased plasma cholesterol levels (by 35–40%), similar to the total cholesterol reduction observed in mice completely lacking PCSK9 (−40–50%).^67,68^

However, the size of the commonly used Cas9 (4.1 kb) from *Streptococcus pyogenes* (SpCas9) limits its utility for basic research and therapeutic applications that use the highly versatile adeno-associated virus (AAV) delivery vehicle. Recently, it was reported smaller Cas9 orthologues from *Staphylococcus aureus* (SaCas9) can edit the genome with efficiencies similar to those of SpCas9, while being more than 1 kb shorter.^107^ When SaCas9 and its single guide RNA expression cassette were packaged into a single AAV vector the authors were able to effectively target the Pcsk9 gene in mouse liver. Within one week of injection, >40% gene modification was observed, accompanied by almost complete absence of immunoreactive Pcsk9 in circulation and a ∼50% reduction in total cholesterol levels, without apparent liver toxicity at one to four weeks after AAV administration.^107^

Obviously, both studies used either adenoviral- or AAV-induced silencing technology, and this viral approach is not yet suitable for human patients, but the reduction of liver PCSK9 by this technology has now been proven. Other methods of delivery of CRISPR-Cas9 and its guide using nanoparticles might become more sophisticated to allow for clinical trials. Furthermore, both studies were short term and were only done in mice. So, the long term benefits and safety associated with the liver-targeted silencing PCSK9 by this technology would have to be proven beyond doubt before it becomes widely used in clinics.

## Conclusons and future directions

This small review presents the many facets of PCSK9 and its biology, concentrating only on its ability to enhance the degradation of the LDLR. However, PCSK9 has been shown to target other members of the LDLR-family including the VLDLR and ApoER2,^108,109^ and can affect the levels of its targets in other tissues than liver, such as small intestine, pancreas and adipose tissue.^7^ Much remains to be unravelled regarding the cellular trafficking of PCSK9 together with its targeted receptors, its possible interactome web, and its binding to other proteins. This is a very exciting period in the field of dyslipidemia, where thanks to new PCSK9-silencing therapies, LDLc levels were lowered to unprecedented levels, reaching almost 0.4 mM. This is good news for hypercholesterolemic patients who do not reach target levels of LDLc with the available medications, cannot tolerate statins, or who experience painful side effects with statins such as myalgia. Even homozygote FH-1 patients that have minimal LDLR activity can now be treated with PCSK9 mAbs with a spectacular ∼30% decrease in circulating LDLc,^110^ thereby giving a much better quality of life that is less dependent on the use of bi-weekly sessions to clear LDLc from their blood using special apheresis dialysis columns.

Although the outcomes of the various ongoing phase III clinical trials using PCSK9-mAbs will not be known until 2018, early signs indicate that this treatment results in a ∼50% reduction in cumulative cardiovascular events within 1 year of treatment.^111,112^ Finally, the fact that PCSK9 is inactivated by some proteases, such as Furin,^52,53^ might open new strategies to enhance this inactivation mechanism and thus lower the levels of active PCSK9. The future will tell which strategies targeting PCSK9, other than mAbs, will find their way in dyslipidemia and cardiology clinics throughout the world and result in affordable and safe treatments.
